# Spatiotemporal variation of chasmogamy and cleistogamy in a native perennial grass: fecundity, reproductive allocation and allometry

**DOI:** 10.1093/aobpla/plad020

**Published:** 2023-04-29

**Authors:** Gregory P Cheplick

**Affiliations:** Biology Program, Plant Sciences Subprogram, City University of New York, New York, NY 10016, USA

**Keywords:** Allometry, chasmogamy, cleistogamy, cleistogene, *Danthonia compressa*, light, mixed-mating system, perennial grass, reproductive allocation, year-to-year variation

## Abstract

It is difficult to assess the relative variability or stability of chasmogamous (CH) and cleistogamous (CL) reproduction in perennial herbs with mixed mating because long-term data in natural populations are unavailable. Here, the aim was to quantify and compare spatial (between-habitat) and temporal (among-year) variation in CH and CL reproduction over 5 years in two subpopulations of the native perennial grass *Danthonia compressa*. This species produces CH spikelets on terminal panicles in early summer, while axillary CL spikelets, including a basal cleistogene, mature into the autumn. Flowering tillers were collected from a sunny woodland edge and an adjacent shady interior habitat for 5 consecutive years (2017–21). Seed set, fecundity, seed mass and biomass allocation were recorded for the two floral types along with tiller vegetative mass. Bivariate line fitting was used for allometric analysis of CH and CL fecundity. Seed set, fecundity, mass per seed and allocation to seeds differed between floral types and showed significant variation between habitats and among years. Seed set and fecundity in CH panicles were greater than that of axillary CL panicles in most years. Tiller mass positively affected axillary CL seed production and mass of the basal cleistogene. Fecundity and allocation among years were more variable for CH compared to CL reproduction. High seed set and fecundity of CH spikelets suggest that pollination does not limit reproduction via chasmogamy. Late maturation of axillary CL spikelets provides additional fecundity, especially in larger plants along sunny woodland edges. The heavy cleistogene at the tiller base could be important to population persistence, analogous to the axillary bud bank of other perennial grasses that are not cleistogamous. The spatiotemporal stability of CL reproduction underscores the ecological significance of cleistogamy to reproductive fitness.

## Teaser

The native grass *Danthonia compressa*produces flowers that open and can outcross in summer plus closed, self-fertile flowers in autumn within leaf sheaths. To assess variation among habitats and years, tillers were collected from two populations in the Catskill Mountains, New York State, for 5 years; seed production (fecundity) and mass were recorded for both floral types. Fecundity and seed mass varied significantly among years and between sunny or shady habitats. Reproduction was much more variable among years for open-pollinated flowers which showed higher fecundity, but lower seed mass, that self-fertile flowers. Late-season maturation of seeds in self-fertile flowers provides additional fecundity, especially in larger plants along sunny woodland edges where this species often occurs. Large basal seeds in sheath-enclosed, self-fertile flowers allow population persistence, similar to buds in other perennial grasses. Stability of seed production within self-fertile flowers underscores the importance of this reproductive mode for this species

## Introduction

Characterization of the spatial and temporal extent of intraspecific variation in phenotypic traits important to fitness is necessary to reveal possible selection pressures responsible for the evolution of plant life histories (Venable 1984; Adler *et al.* 2014; Cheplick 2015a; Shipley *et al.* 2016; Westerband *et al.* 2021). Intraspecific variation occurs not only among populations, but also between and within individuals. Within- and between-population variations are important to continue phenotypic evolution (Bradshaw 1991; Mazer and Damuth 2001; Picó *et al.* 2021) in response to heterogeneous habitats. Within-individual variation reflects the modular construction of plants in which repeating subunits or metamers (White 1984) comprise the individual (Vuorisalo and Mutikainen 1999; Herrera 2009, 2017). The contribution of modularity to within-individual variation is illustrated by the many floral and vegetative traits that vary across the reiterated structures of a plant (reviewed by Herrera 2009). This variation can affect key traits important to fitness such as individual fecundity (Herrera 2017). For example, varying the number of branches and modules that comprise the plant body can affect seed maturation and maximize fecundity during a growing season, especially when inflorescences form in the leaf axils of developing modules (Smith 1984; Cheplick 2002, 2015b).

Dimorphic cleistogamy is one example of potentially adaptive within-individual variation whereby both open-pollinated chasmogamous (CH) flowers and closed, self-fertilized cleistogamous (CL) flowers are produced by the same individual (Culley and Klooster 2007). Cleistogamous species often produce morphologically distinct CH and CL flowers that develop in different locations on the plant body (Campbell *et al.* 1983; Ellstrand *et al.* 1984; Cheplick 2006) and/or mature seeds at different times within a growing season (Schemske 1978; Schoen 1984; Berg and Redbo-Torstensson 1998; Oakley *et al.* 2007; Sternberger *et al.* 2020).

Cleistogamous reproduction has traditionally been viewed as a form of reproductive assurance that provides a way for plants to mature many seeds at a low energetic cost under conditions of pollinator scarcity (Darwin 1877; Kalisz *et al.* 2004; Goodwillie *et al.* 2005; Eckert *et al.* 2006; Veena and Nampy 2019; Delgado-Dávila and Martén-Rodríguez 2021) or stressful abiotic conditions (Campbell *et al.* 1983; Schoen 1984; Le Corff 1993; Cheplick 2007). Indeed, the low cost of producing CL flowers and their high seed set (Schemske 1978; Schoen 1984; Redbo-Torstensson and Berg 1995; Winn and Moriuchi 2009; Hesse and Pannell 2011; Veena and Nampy 2019; Seguí *et al.* 2021), and the absence of significant inbreeding depression in many selfing species (Culley 2000; Winn *et al.* 2011; Ansaldi *et al.* 2019; Delgado-Dávila and Martén-Rodríguez 2021), argues forcibly for an evolutionary advantage of CL under many conditions. Nevertheless, exclusively CL species are not known, suggesting some advantage to the additional production of CH flowers (Oakley *et al.* 2007), especially when environmental conditions vary greatly in space and time (Schoen and Lloyd 1984).

In nature, heterogeneous habitats with variable abiotic factors such as availability of light and soil moisture profoundly affect the relative proportions of fruits and seeds matured in CH and CL flowers (Wilken 1982; Bell and Quinn 1987; Masuda and Yahara 1994; Cortés-Palomec and Ballard 2006; Cheplick 2007; Ranua and Weinig 2010). In cleistogamous species, there can also be position-dependent effects on CH and CL reproduction within an individual (Ellstrand *et al.* 1984; Cheplick and Sung 1998) as well as habitat-specific differences among populations and year-to-year variation in the production of CH and CL flowers, fruits and seeds and the allocation of biomass to them (Jasieniuk and Lechowicz 1987; Cortés-Palomec and Ballard 2006; Cheplick 2007; Ranua and Weinig 2010; Furukawa *et al.* 2020). Note that, although the terms cleistogamy and chasmogamy technically express particular floral conditions (Lord 1981), here the modifiers ‘CH’ and ‘CL’ are extended to caryopses/seeds matured in the floral types, as applied by others (Campbell *et al.* 1983; Clay 1983*b*). The relative production of CH and CL propagules and the allocation of resources to them often vary in space and time with changing environmental conditions that affect plant size (Wilken 1982; Berg and Redbo-Torstensson 1998; Diaz and Macnair 1998; Cheplick 2007). CH reproduction has sometimes been found to be opportunistic and associated with favourable conditions that result in larger plants with more resources available, while CL reproduction predominates in less favourable environments (Schemske 1978; Schoen and Lloyd 1984; Ansaldi *et al.* 2018; Sternberger *et al.* 2020). However, it is difficult to assess the relative variability (or stability) of CH and CL reproduction of perennial species in diverse habitats because long-term data on reproductive parameters in natural populations are unavailable for most CL species. The present research attempts to address this issue by presenting data on the CH and CL components of reproduction in two established subpopulations of a native perennial grass sampled over a 5-year period. One subpopulation occupied a sunny, woodland edge habitat, while the other occupied an adjacent, shady interior habitat.

Specific questions and predictions addressed were (i) do the primary components of reproduction (number of florets, seed set, fecundity, seed mass and allocation) differ between CH and CL modes and how do these components vary with habitat conditions and among years? Based on previous research cited earlier, it was predicted that the sunnier edge habitat would be more favourable to CH reproduction and that CL reproduction would predominate in the less favourable shadier habitat beneath the woodland canopy. Year-to-year variation in spring and summer temperature and precipitation were expected to contribute to temporal variation in the components of CH and CL reproduction similarly in the two habitats. (ii) Do the components of CH and CL reproduction show allometric relationships to the size of the tillers on which seeds mature? Based on prior research on CH–CL species, it was predicted that larger tillers would show greater reproduction by CH relative to CL (i.e. higher CH fecundity).

## Materials and Methods

### Ecology and botany of *Danthonia compressa*

*Danthonia compressa* is a perennial bunchgrass that exhibits dimorphic cleistogamy in which both CH and CL flowers are made on the same individual (Culley and Klooster 2007), a situation found in other members of the genus (Chase 1918; Weatherwax 1928; Dobrenz and Beetle 1966; Clay 1983*a*). The production of CH and CL spikelets in *D. compressa* is separated in both time and space. In the temperate climate of southern New York State, flowering tillers bearing CH spikelets on terminal panicles are found in late June and mature caryopses are present by late July. Typically only 1–4 flowering tillers are made per reproductive adult in the subpopulations examined here (mean ± standard error [SE] = 1.7 ± 0.3 flowering tillers for 35 plants in a woodland edge with a mean total number of tillers = 22.3 ± 1.5; Cheplick 2023).

The grass phytomer is the modular unit of tiller morphology and consists of a leaf blade and sheath, node, internode and axillary bud (Briske and Derner 1998; Peretta *et al.* 2011). The bud in each phytomer of a flowering tiller of *D. compressa* gives rise to a small axillary panicle with CL spikelets that later (mid-September) mature seeds within the leaf sheath. In addition, the lowermost 1–2 phytomers each mature a single large caryopsis, the cleistogene (Chase 1918; Dyksterhuis 1945; Dobrenz and Beetle 1966; Campbell *et al.* 1983). Due to their functional identity to seeds, caryopses will simply be referred to as ‘seeds’, with ‘cleistogene’ referring to the CL seed in the lowermost phytomer and ‘axillary seeds’ referring to CL seeds at the additional phytomers along a flowering tiller [see **Supporting Information—Fig. S1**].

Note that as one proceeds from the basal phytomer towards the uppermost phytomer bearing the emergent panicle on a flowering tiller, axillary CL seeds become more numerous but smaller and lighter in mass. This pattern has been described in other grass species with axillary cleistogamy (Cheplick and Clay 1989; Cheplick 1996; Lerner *et al.* 2008). In *D. Compressa*, there are 4–7 phytomers along a flowering tiller. Although self-fertilization routinely occurs in basal and axillary CL spikelets, potentially out crossed CH spikelets on emergent terminal panicles are also capable of self-fertilization in CH–-CL grasses (Brown 1952; Cheplick and Quinn 1986; Philipson 1986; Murphy and Aarssen 1996). In *D. compressa* and other perennial grass species with mixed mating, seeds from CL florets are often larger, show greater germination or produce more vigorous seedlings than seeds from CH florets (Dobrenz and Beetle 1966; Campbell *et al.* 1983; Clay 1983*b*; Bell and Quinn 1985; Call and Spoonts 1989; Cheplick 2023).

*Danthonia compressa* ranges from North Carolina in the USA northward to southern Canada and is restricted to eastern North America (Darbyshire 2003). It is usually found in open or partly shaded areas along woodland edges, but can also occur beneath relatively shady woodland canopies.

### Study site and habitat conditions

The two adjacent habitats chosen for long-term monitoring of *D. compressa* reproduction were in the northern portion of the Catskill Mountains at 527 m in southern New York State (42° 26ʹ 6.87″ N, 74º 49ʹ 47.61″ W). This secondary-growth woodland is dominated by sugar maple (*Acer saccharum*), red maple (*Acer rubrum*), American beech (*Fagus grandifolia*) and white birch (*Betula papyrifera*). The smaller trees, hop hornbeam (*Ostrya virginiana*) and black cherry (*Prunus serotina*), are along the edges. Additional information on Catskill forests can be found in McIntosh (1972). Soils are shallow sandy loams with numerous stones derived from glacial till and have an average depth of 56.6 cm (Johnson 2013).

Mean annual precipitation in the region was 116 cm, and mean annual maximum and minimum temperatures were 12.9 and 0.9 °C, respectively, for the period 1991–2020 at the nearest US National Weather Station, 15 km from the study site (Climate Data Online, National Centers for Environmental Information, National Oceanic and Atmospheric Administration [www.ncdc.noaa.gov]). Note that this source was also used to obtain daily temperature and precipitation data for the May through September growing season in the 5 years of this study (2017–21). Growing season length in the Catskills region is 123–152 days from May through October (Anandhi *et al.* 2013).

The two contrasting adjacent habitats designated ‘edge’ and ‘interior’ contained subpopulations of *D. compressa* separated by 15–30 m. About 50–60 individuals were along the woodland edge, an area about 6 m wide and not under a canopy of trees. Direct sunlight impacted this habitat for 4–6 hours per day. In contrast, the interior habitat contained about 75–80 individuals completely under a canopy of trees, about 6–10 m in from a woodland/field boundary (map in Cheplick 2023). Following canopy leaf expansion in late May, photosynthetic photon flux at mid-day in the interior was ~23 % of the light level in the edge (data in Cheplick 2023) and was only available as sun flecks and filtered sunlight. However, in late August 2020, the fourth year of this study, a lightning strike split a large (76.0 cm dbh) sugar maple tree about 2 m up the trunk in the interior habitat. Subsequently, two branches fell and opened up the canopy above the *D. compressa* subpopulation. It was expected that this rare, lighting-caused canopy gap would alter environmental conditions (Yanoviak *et al.* 2015), especially light availability for plants the next year, the fifth year of the tiller collection.

During May to August of 2020, mean (±SE) volumetric water content was 10.2 (±0.6) % in the edge compared to 13.4 (±0.5) % in the interior (45 readings per habitat, *F* = 27.5, *P* < 0.001; Cheplick 2023).

### Flowering tiller collection

Each terminal panicle bearing mature CH seeds that had not yet dispersed was covered with an envelope and the culm excised at the flag leaf. Panicles were collected haphazardly from 15 individuals per habitat between 20 and 27 July in each of the 5 years of this study (2017–21). Each year, sampled individuals were at least 1 m apart and covered the full extent of the same area occupied by each subpopulation. These flowering tillers were tagged with a twist-tie metallic tag at the base. In each year between 6 and 21 September, depending on when tillers exhibited signs of senescence, the complete tiller containing sheath-enclosed CL seeds [see **Supporting Information—Fig. S1**] was excised at ground level and placed into a paper bag. All separate panicles and tillers were stored dry at room temperature until further processing.

### Primary variables

Every year before drying the plant tissues, the following data were collected for each flowering tiller: (1) number of phytomers, (2) number of CH and CL seeds (= fecundity), and (3) number of empty CH and CL florets that had not matured a seed. Then, all tiller components retained separately by individual and habitat-of-origin were dried in a 60 °C convection oven to constant mass. Dry mass was obtained to 0.1 mg for (4) vegetative structures (leaves and culm), (5) terminal panicle (*sans* CH seeds), (6) CH seeds, (7) axillary CL seeds and (8) the basal CL seed (= cleistogene) [see **Supporting Information—Raw Data**].

### Derived variables

Because a single floret contains one ovule that matures into a single seed, the proportion of CH florets that set seed on a terminal panicle was the (number of CH seeds)/(number of empty CH florets + number of CH seeds). Note that for a grass, this is essentially equal to a seed/ovule ratio or a fruit/flower ratio (Wiens 1984). In a similar manner, the seed set in axillary CL florets was the (number of axillary CL seeds along the tiller)/(number of empty CL florets + number of CL seeds). For both floral types, the total number of florets made by a tiller provides an estimate of the initial investment to CH and CL and is the sum of the number of empty florets plus the number that contains a seed. Because a cleistogene was present at the basal node of all flowering tillers and results from self-fertilization within a single floret (Chase 1918; Weatherwax 1928), the seed set of basal florets was 100 %.

Mass per seed was the collective mass of all CH and axillary CL seeds made by a tiller divided by the total number of CH or axillary CL seeds, respectively. The proportion of the total fecundity per tiller due to cleistogamous reproduction was (number of CL seeds)/(number of CH seeds + number of CL seeds).

Reproductive allocation (RA) was expressed as the dry mass of all CH or CL seeds (including the cleistogene) relative to tiller vegetative mass (Cheplick 2020). Allocation to CL seeds (RA_CL_) is the sum of axillary and basal seed mass relative to vegetative mass. The allocation to CH seeds only is designated RA_CH1_. However, because some researchers maintain that the reproductive supporting structures of flowers, fruits and seeds should be included in the calculation of RA (Thompson and Stewart 1981; Reekie 1999), a second expression of allocation to CH was calculated that included the mass of the culm and panicle branches that bore the CH spikelets and seeds above the flag leaf (Wilson and Thompson 1989). This was RA_CH2_ = (CH seed mass + panicle mass)/tiller vegetative mass.

### Data analysis

All analyses used Version 9.4 of the Statistical Analysis System, University Edition (SAS Institute, Cary, NC).

To control for variation in tiller size among years and habitats, a three-way analysis of covariance (ANCOVA) was used for the number of florets, seed set proportion, fecundity (= number of seeds) and mass per seed. The covariate was the vegetative mass of the tiller; sources of variation (all fixed) were year, habitat, and floral type and their interactions. RA which incorporates tiller mass into its calculation was analysed by three-way ANOVA without a covariate. To comply with the assumption of homogeneity of variances (Dowdy *et al.* 2004), seed set and allocation were arcsine, square-root transformed while mass per seed was log-transformed.

The number of phytomers, cleistogene mass and the proportion of total fecundity due to CL seed production were analysed by two-way ANCOVA, again with tiller mass as the covariate. Sources of variation were year, habitat and their interaction. Number of phytomers and cleistogene mass were log-transformed to comply with ANCOVA assumptions.

Following ANCOVAs, least squares means were computed and used for multiple comparisons of the adjusted means (Dowdy *et al.* 2004; Cai 2014). To examine temporal variation among CH and CL components of reproduction, coefficients of variation (CV) were calculated as (standard deviation/mean) ×100 using the grand means of each sampled group of tillers over the 5 years in each habitat and the standard deviation among the individual yearly means; afterwards, 95 % confidence intervals were determined using the SE of the CV estimate (Dowdy *et al.* 2004). However, caution should be exercised in interpreting the confidence intervals because this approach assumes a normal distribution of CV values. The CV can be a useful metric for temporal variability when there are no zero values and the specific chronological sequence is not of interest (Fernández-Martínez and Peñuelas 2021). Note that the CV as calculated here represents a population-level estimate of the variability in the means of the phenotypic traits among years (Herrera 1998).

To complement the ANCOVA model in which tiller vegetative mass was included as a covariate to analyse reproductive variables, the bivariate line-fitting method using standardized major axes (Warton *et al.* 2006) was used for the allometric analysis of fecundity by CH and CL reproduction in each year. Also known as reduced major axis (Model Type II) regression, this analysis expresses the relationship logY_2_ = logβ_RMA_ + α_RMA_ logY_1_, where Y_2_ and Y_1_ are the bivariates, β_RMA_ is the *y*-intercept, and α_RMA_ is the slope (Niklas 2004). Here Y_2_ is the number of CH or axillary CL seeds matured on a tiller (i.e. fecundity) and Y_1_ is the vegetative dry mass of the tiller in either habitat. Although reproductive allometry can also be examined as the biomass of reproductive parts relative to vegetative mass (e.g. Weiner *et al.* 2009), fecundity can be useful in allometric analysis (Aarssen and Taylor 1992; Shipley and Dion 1992; Cheplick 2020) as it allows separation of the number of seeds from the collective mass of seeds produced. Fecundity and mass per seed may better estimate reproductive fitness via cleistogamy and chasmogamy as separate variables. In addition, fecundity and the collective mass of seeds of a tiller were always tightly correlated: for all years, the correlations between the collective mass of CL seeds and the number of CL seeds were all highly significant (*r*^2^ from 0.79 to 0.91, all *P* < 0.0001), as were all correlations between the collective mass of CH seeds and number of CH seeds (*r*^2^ from 0.82 to 0.95, all *P*< 0.0001). Because the allometry of fecundity involved the analysis of 5 years for two seed types (10 regressions), Bonferroni correction was applied to interpret statistical significance (*P*) at 0.05/10 = 0.005.

To explore how modular construction of a flowering tiller could differentially affect fecundity by CH or CL, regression analyses were performed for tillers from each habitat across all years. The number of CH or CL seeds was regressed onto the number of phytomers of a tiller, both log-transformed. Mean (±SE) number of seeds of each type was calculated for tillers comprised of 4–7 phytomers in each habitat.

## Results

The number of florets, seed set, fecundity and mass per seed depended significantly on floral type (CH vs. CL) and varied temporally (among years) and spatially (between habitats; Table 1). In addition, tiller vegetative mass had a significant positive effect on the number of florets and seeds produced (Table 1).

**Table 1.** ANCOVAs of seed set proportion (arcsine, square-root transformed), number of florets and seeds per flowering tiller, mass per seed (log-transformed), and ANOVA of allocation to seeds (arcsine, square-root transformed) for chasmogamous (CH) and cleistogamous (CL) components of reproduction in *Danthonia compressa*.

Source of variation	Number of florets			Seed set		Number of seeds		Mass per seed		Allocation	
	d.f.	M.S.	*F*	M.S.	*F*	M.S.	*F*	M.S.	*F*	M.S.	*F*
Year	4	646.9	20.86^***^	0.9513	24.91^***^	339.3	11.88^***^	0.0434	5.97^***^	0.0294	5.47^***^
Habitat	1	199.0	6.42^**^	0.2215	5.80^*^	254.3	8.90^**^	0.0567	7.79^**^	0.0012	0.23
Type	1	177.9	5.73^*^	0.7802	20.43^***^	380.8	13.33^***^	3.5438	487.46^***^	0.1541	28.63^***^
Year × habitat	4	43.7	1.41	0.1544	4.04^**^	115.0	4.03^**^	0.0142	1.95	0.0261	4.84^***^
Year × type	4	590.8	19.05^***^	0.1436	3.76^**^	384.6	13.46^***^	0.0094	1.30	0.0536	9.95^***^
Habitat × type	1	227.1	7.32^**^	0.2794	7.32^**^	27.0	0.95	<0.0001	<0.01	0.0096	1.78
Year × hab. × type	4	50.8	1.64	0.0829	2.17	73.1	2.56	0.0104	1.44	0.0110	2.04
Vegetative mass	1	1364.9	44.0^***^	0.0041	0.11	791.8	27.72^***^	0.0198	2.73	—	—
Error	279	31.0		0.0382		28.6		0.0073		0.0054	

Flowering tillers were from woodland edge or interior habitats during summer in 5 successive years (2017–21). ‘Type’ refers to CH versus CL. For the first four variables, vegetative mass of the flowering tiller was entered into the complete model as a covariate.

^*^ *P*< 0.05;

^**^ *P*< 0.01;

^***^ *P*< 0.001.

### Number of florets

Year and the year by floral type interaction had highly significant effects on floret number (Table 1); temporal variation for CH floret production was over two times as great as that for CL floret production (Table 2). Investment in CH floret production was greatest in the sunny edge, comprising 54.2 % of the total florets made by a flowering tiller across the 5 years, but in the shady interior equal numbers of CH and CL florets were produced (18 of each per tiller) [**see****Supporting Information—Table S1**].

**Table 2.** Coefficients of variation (%) ± 95 % confidence intervals across the 5 years of *Danthonia compressa* tiller collection for components of chasmogamous (CH) and cleistogamous (CL) reproduction.

Trait	Edge		Interior	
	CH	CL	CH	CL
Number of florets	28.96 ± 8.03	12.58 ± 3.49	33.82 ± 9.37	16.28 ± 4.51
Seed set	16.43 ± 4.55	9.64 ± 2.67	18.12 ± 5.02	17.86 ± 4.95
Number of seeds	25.74 ± 7.13	17.71 ± 4.91	38.98 ± 10.80	25.17 ± 6.98
Mass per seed	11.01 ± 3.05	4.25 ± 1.18^*^	7.95 ± 2.20	9.93 ± 2.75^*^
Allocation	26.51 ± 7.35	12.69 ± 3.52	48.09 ± 13.33	24.41 ± 6.77

Coefficients are shown separately for edge and interior habitats.

^*^Axillary CL seeds only; separate coefficients for basal cleistogene mass were 13.75 ± 3.81 in the edge and 20.71 ± 5.74 in the interior.

### Seed set

**Figure 1.**
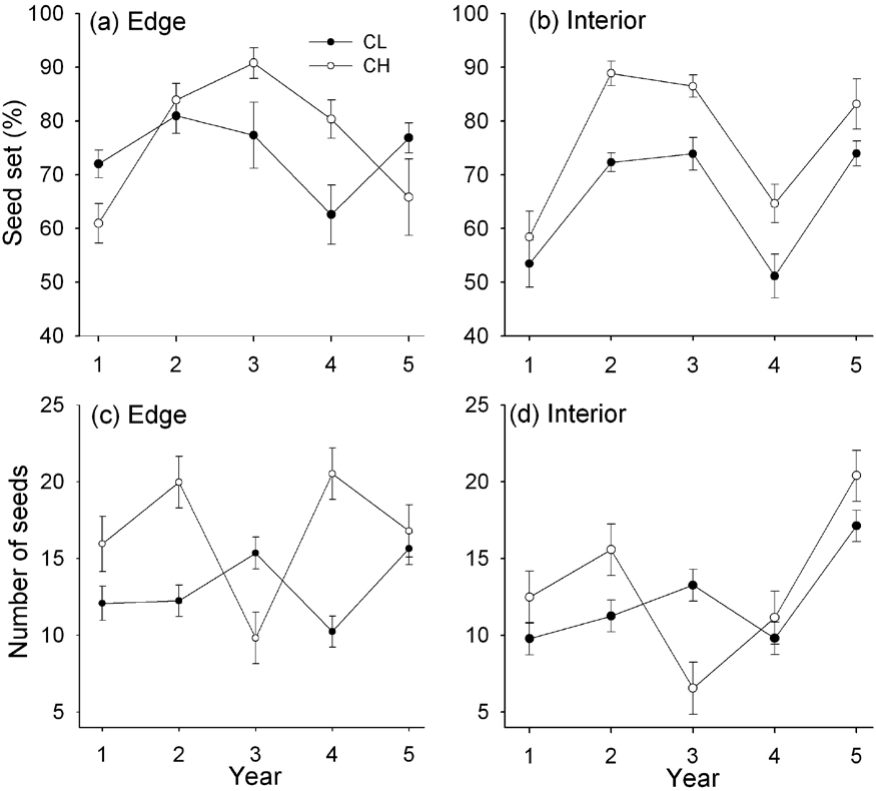
Mean (±SE) seed set percentage in CH and axillary CL florets on flowering tillers in the edge (**A**) and interior habitat (**B**), and mean (±SE) number of CH or CL seeds (fecundity) in the edge (**C**) and interior habitat (**D**), over the 5 years of the study.

The percentage of seeds set in CH florets was greater than that in axillary CL florets in most years in both habitats (Fig. 1A and B). CH seed set was about 76 % in both habitats, while CL seed set was higher in the edge (74 %) relative to the interior (65%; [**see****Supporting Information—Table S1**]. CH seed set was greatest (>80 %) in Years 2 and 3 in both habitats (Fig. 1A and B) and in Year 4 in the edge (Fig. 1A), all years during which temperatures were above normal in July when CH seeds mature [**see****Supporting Information—Fig. S2**]. Vegetative mass did not affect the seed set of either floret type. Temporal variation was greater for CH than CL seed set in the edge (Table 2) but was similar across years in the interior (Fig. 1B).

### Fecundity

Fecundity varied greatly among years and floral type (Fig. 1C and D) and depended significantly on tiller mass (Table 1). Fecundity of CH florets showed a pronounced dip in Year 3 which did not occur for CL florets (significant year × type interaction in Table 1). The precipitation in July when CH seeds mature was lowest in Year 3 [**see****Supporting Information—Table S2**]. In the edge, least squares mean ± SE number of seeds per tiller was 16.6 ± 1.9 for CH and 13.1 ± 1.0 for CL across the 5 years. In the interior, fewer seeds were made by CH (13.2 ± 2.3) but the number of CL seeds (12.2 ± 1.4) was similar to the number made in the edge [**see****Supporting Information—Table S1**]. As expected, following the opening of the canopy of the interior by a lightning strike in August of Year 4, there was a significant increase in the fecundity of both CH and CL in Year 5 (Fig. 1D). Temporal variation in fecundity was much greater for CH relative to CL reproduction (Table 2).

**Figure 2.**
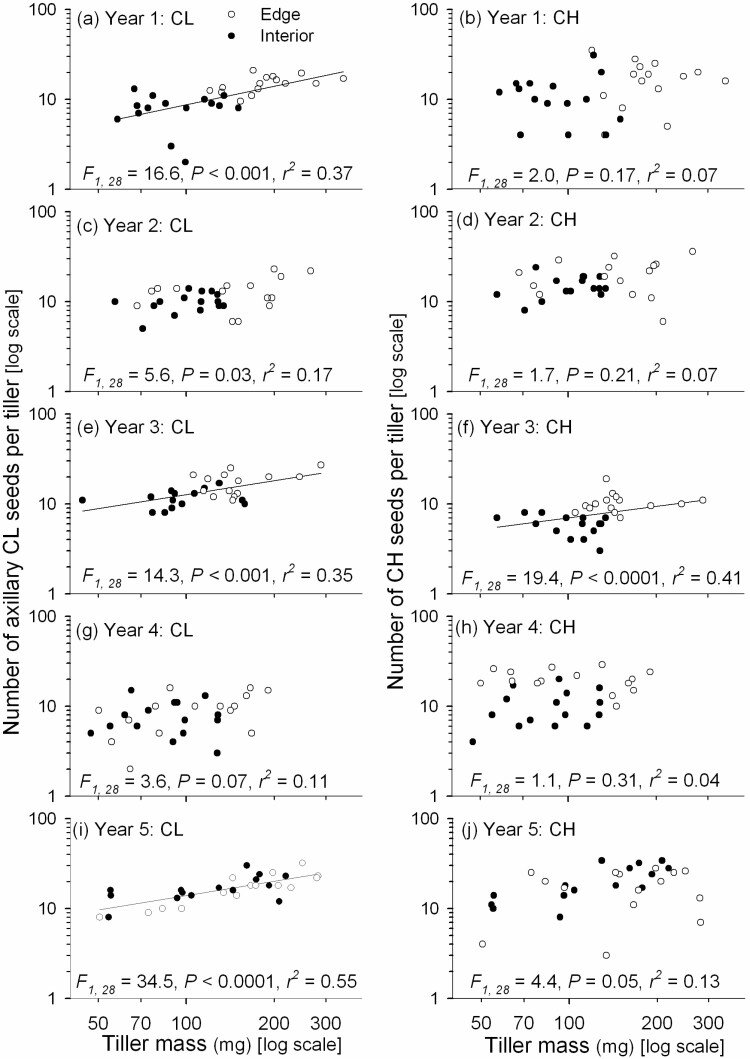
The number of axillary CL seeds (**A**, **C**, **E**, **G**, **I**) and number of CH seeds (**B**, **D**, **F**, **H**, **I**) regressed onto vegetative mass of the flowering tiller for each of the 5 years of the study. Only statistically significant (after Bonferroni correction) regression lines are shown.

Analysis of the allometry of fecundity revealed that the significant effect of tiller mass on seed production (Table 1) was mostly manifest in CL reproduction. An increase in CL fecundity with tiller mass was highly significant (*P* < 0.001) in Years 1, 3 and 5 (Fig. 2) and close to significant in year 2 (*P* = 0.03). In contrast, CH fecundity showed an increase with tiller mass only in Year 3 (*P* < 0.001; Fig. 2). Slopes of the significant standardized major axis regressions were mostly close to 1.0, indicating isometry of fecundity with tiller mass [see **Supporting Information—Table S3**].

**Figure 3.**
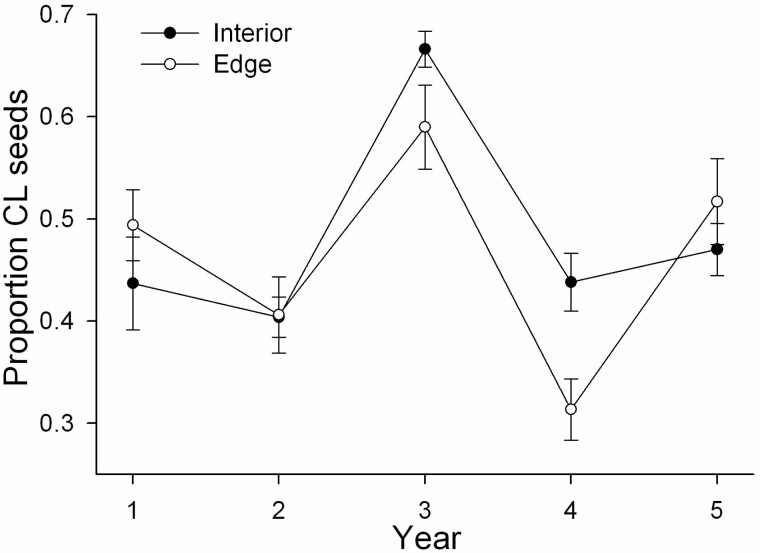
Mean (±SE) proportion of the total fecundity per tiller due to CL seed production in the edge (**A**) and interior habitat (**B**) over the 5 years of the study.

The proportion of the total fecundity per tiller due to CL seed production depended greatly on year (*F*_4,138_ = 15.5, *P* < 0.0001), but was not significantly affected by habitat (*F*_1,138_ = 2.5, *P* = 0.11) or tiller mass (*F*_1,138_ = 2.3, *P* = 0.13). However, there was a significant interaction of year by habitat (*F*_4,138_ = 3.2, *P* = 0.02). In Years 3 and 4, the proportion of CL seeds was greater in the shady interior (mean ± SE = 0.67 ± 0.02 and 0.44 ± 0.03 in Years 3 and 4, respectively) compared to the sunny, edge habitat (0.59 ± 0.04 and 0.31 ± 0.03 in Years 3 and 4, respectively; Fig. 3). The dip in CH fecundity in Year 3 in both habitats without a concurrent reduction in CL fecundity (Fig. 1C and D) is reflected in the much higher proportion of CL reproduction that year in both habitats (Fig. 3).

The number of phytomers on a flowering tiller varied from four to seven and seed production by axillary CL was significantly greater in tillers with more phytomers in both habitats [see **Supporting Information—Table S4**]. In contrast, CH seed production was not related to the number of phytomers in the edge habitat; however, in the light-limited interior, CH fecundity was significantly greater in tillers with more phytomers.

### Mass per seed

**Figure 4.**
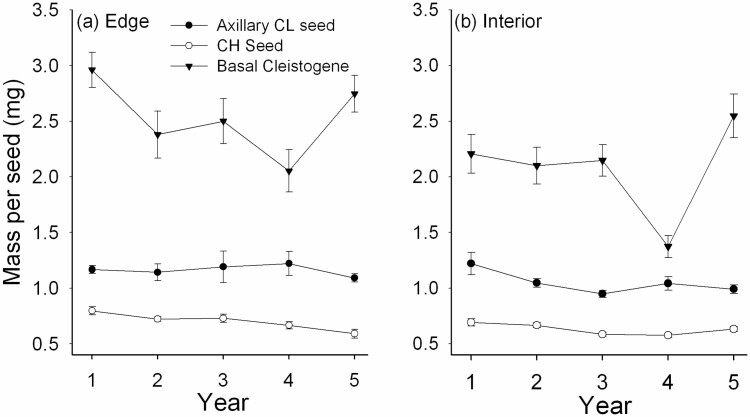
Mean (±SE) mass per CH seed, axillary CL seed, and basal seed (cleistogene) in the edge (**A**) and interior habitat (**B**) over the 5 years of the study.

The mass per axillary CL seed was always much greater than the mass per CH seed (Fig. 4) and floral type was highly significant in the ANCOVA (Table 1). Tiller mass did not affect mass per axillary CL or CH seed. Although there was significant temporal and spatial variation (Table 1), the mean mass per seed was the least variable among the years of the traits examined (Table 2). Averaged across the 5 years, in the edge, mean ± SE mass per seed for CH, axillary CL, and cleistogene were 0.70 ± 0.03, 1.16 ± 0.02 and 2.53 ± 0.15 mg, respectively, while mass per seed was reduced in the interior [see **Supporting Information—Table S1**].

In a separate ANCOVA of cleistogene mass, year (*F*_4,138_ = 8.7, *P* < 0.0001), habitat (*F*_1,138_ = 6.1, *P* = 0.01) and tiller mass (*F*_1,138_ = 16.0, *P* < 0.0001) were all significant sources of variation. The latter effect showed that heavier tillers produced heavier cleistogenes in both habitats [see **Supporting Information—Fig. S3**].

### Reproductive allocation

**Figure 5.**
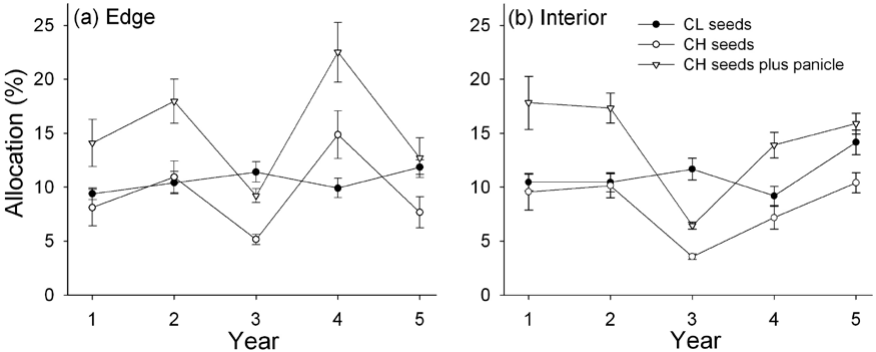
Mean (±SE) percent allocation of tiller mass to CL seeds, CH seeds, or total CH reproduction (seeds plus panicle) in the edge (**A**) and interior habitat (**B**) over the 5 years of the study. Note the relative temporal stability of CL compared to CH allocation (coefficients of variation across years are in Table 2).

RA, assessed as the collective mass of either CH or CL seeds made on a flowering tiller relative to tiller vegetative mass, was significantly affected by year and floral type (Table 1). Allocation to CL seeds (RA_CL_), including the heavy cleistogene, showed low variation among years (Fig. 5; Table 2). RA_CL_ was 10.6 ± 0.4 % in the edge (all RA data are means ± SE) and 11.1 ± 0.5 % in the interior habitat [**see****Supporting Information—Table S1**]. Across years, RA_CL_ ranged from 9.2 ± 0.9 % in the interior in Year 4 to 14.2 ± 1.1 % in the interior in Year 5 (Fig. 5B).

Allocation to CH seeds (RA_CH1_) showed much greater variation among years: CVs in RA_CH1_ were about double that of RA_CL_ (Table 2). RA_CH1_ averaged 9.4 ± 0.8 % in the edge and 8.2 ± 0.6% in the interior habitat [see **Supporting Information—Table S1**]. Across years, RA_CH1_ ranged from 3.6 ± 0.3 % in the interior in Year 3 to 14.9 ± 2.2 % in the edge in Year 4 (Fig. 5).

Inclusion of terminal panicle mass as part of the allocation to CH (RA_CH2_) did not change the pattern of allocation among years shown by RA_CH1_ (Fig. 5) and the statistical significance of all sources of variation [see **Supporting Information—Table S5**] mirrored that of RA_CH1_ (Table 1). Of course, inclusion of panicle mass increased the values of RA [see **Supporting Information—Table S1**]. Across years, RA_CH2_was consistently greater than RA_CL_, except in Year 3 (Fig. 5).

## Discussion

### Spatiotemporal variation in chasmogamy and cleistogamy

The reproductive variables associated with CH and CL, that is, the number of florets and seeds, mass per seed, and proportional allocation, depended greatly on habitat and the year of tiller collection in the perennial grass *D. compressa*. The modular construction of the flowering tiller allows the maturation of a heavy basal cleistogene plus additional axillary CL panicles enclosed by the leaf sheath of each phytomer late in the growing season after terminal panicles with CH seeds have already matured and dispersed. Cleistogamy may, therefore, be highly advantageous in *D. compressa* because it provides a reproductive assurance and is not as affected by environmental conditions among habitats or across years as CH reproduction. Given that about 75 % of the grass species with cleistogamy are caespitose (Campbell *et al.* 1983), the dense clustering of tillers in CL bunchgrasses can not only promote population persistence in competitive environments such as grasslands or successional old fields (Pyke 1990; Rychtecká and Lepš 2020), but the basal cleistogene and axillary CL seeds may also have a similar role to play in population persistence (see *Discussion: Role of the Cleistogene*).

The production of CH and CL florets and seeds by *D. compressa* is separated in time within a single season and in their spatial position on the flowering tiller. The production of CH flowers prior to CL flowers is not a universal feature of CH–CL species (Culley and Klooster 2007), but the late maturation of selfed CL spikelets in *D.compressa* suggests that cleistogamy provides some potential for reproductive assurance in the event of insufficient or failed pollination of the CH flowers (Redbo-Torstensson and Berg 1995; Kalisz *et al.* 2004; Goodwillie *et al.* 2005; Eckert *et al.* 2006; Veena and Nampy 2019). For example, in two herbaceous perennial species examined by Redbo-Torstensson and Berg (1995), production of CL flowers continued late into September and compensated for the loss in seed set due to the lack of fertilization of CH flowers produced earlier in May.

However, the seed set of CH florets on the terminal panicles of *D. compressa* was high in the two subpopulations studied, often exceeding that of the axillary CL florets (Fig. 1A and B). This suggests that wind pollination was not limiting to out crossing by chasmogamy or that CH florets are capable of self-fertilization (Philipson 1986; Murphy and Aarssen 1996). Murphy and Aarssen (1996) reported that seed set on CH panicles of *D. compressa* often exceeded 70 % in the field, a result in agreement with this study (Fig. 1A and B). Schoen (1984) reported a much higher seed set (98 %) in CL compared to CH flowers (23 %), and the latter was much more variable in the perennial grass *Microlaena polynoda*. Berg (2003) speculated that the very high seed-to-ovule ratios found in the CH flowers of four herbaceous species with mixed-mating systems were due to high selfing rates in these flowers and it is suggested that selfing also frequently occurs in the CH florets of *D. compressa* panicles as it does in *D. spicata* (Philipson, 1986), resulting in a high seed set.

In two populations of *D.compressa* in North Carolina which is near the southernmost part of the species’ range (Darbyshire 2003), Clay (1983*a*) reported that about 50 % of the total fecundity was due to seed production by CL. This agrees roughly with the proportion of CL found in the present study, although there was much variation from year to year, with CL ranging from 31 to 67 % of total seed production (Fig. 3).

Pronounced variation in seed set and fecundity in successive years is not unusual in perennial grasses (Smith 1944; Cheplick 2021). In *D. compressa*, environmental differences among annual flowering seasons and between habitats provided spatiotemporal variation that clearly affected reproduction by both CH and CL. For example, greater light availability in the sunnier woodland edge compared to the shadier interior was probably the most important spatial environmental factor in regard to flowering and seed production in CH panicles. Over the 5 years of this study, CH floret production was greatest in the edge and CH fecundity was reduced by 20.5 % in the interior relative to the edge, while CL fecundity was only reduced by 6.9 % in the interior. CL reproduction also showed greater stability among years: relative to CL floret numbers and fecundity, CH reproduction had much greater year-to-year variation (Table 2). These observations suggest CL reproduction is less affected by environmental variation than CH reproduction, although seed production by both floral types increased in Year 5 in the interior habitat after light availability improved following canopy opening by a lightning strike the previous year (Fig. 1D).

Although it is presently unclear why CL is less affected by environmental variation than CH, it is suggested that axillary CL panicles enclosed by phytomer leaf sheaths are better situated to sequester photosynthates from leaves distributed along a flowering tiller compared to emergent CH panicles which are furthest from the leaves and possibly less likely to secure limited photosynthates in years or in habitats where light availability is low. Vascular strands of a phytomer entering an axillary bud (such as one that produces an axillary CL panicle) are derived from the subtending leaf in grasses (Chapman and Peat 1992; Marshall 1996); the partially autonomous physiological units that comprise the bunchgrass life form (Briske and Derner 1998) could constrain the movement of photosynthates within the plant body (Watson and Casper 1984; Hererra 2009). This sectorial construction (Marshall 1996) might then buffer the effects of environmental variation on axillary CL panicle and seed production relative to terminal CH panicles, although this is speculative because the movement of carbon within CL grasses has not yet been investigated. Alternatively, a reserve supply of resources maintained for CL reproduction could help minimize environmental effects and ensure seed production under most conditions, while maternal investment (Lloyd 1980) of additional resources into CH reproduction mostly occurs under favourable conditions (Schoen and Lloyd 1984; Oakley *et al.* 2007). By not using stored reserves to buffer environmental variation for terminal panicle production, maternal investment into reproduction may need to be continually adjusted to the resources available during a growing season (Lloyd 1980) and therefore be more sensitive to changes in environmental conditions over space and time.

The mass per CH or axillary CL seed were both relatively stable across the years (Table 2), but were significantly reduced in the interior habitat, suggesting that attenuated sunlight limits the ability to mature heavy seeds. This was especially evident in the basal cleistogene where mass was reduced by 18.2 % in the interior relative to the edge; comparable seed mass reduction for axillary CL and CH seeds was 9.5 % and 10.0 %, respectively. In addition, while axillary CL and CH seed mass were unrelated to tiller mass, heavier tillers were associated with heavier cleistogenes [see **Supporting Information—Fig. S3**], implying that resource availability was important to the maturation of large basal seeds. Note that, in contrast to data presented by Clay (1983*a*) and Murphy and Aarssen (1996) showing similar mean mass of CH and CL seeds in *D. compressa*, the present study found that axillary CL seeds and cleistogenes especially were both much heavier than CH seeds from terminal panicles in either habitat (Fig. 4). The greater mass of CL seeds in these subpopulations of *D. compressa* results in larger seedlings compared to those from CH seeds and is likely to be important to seedling establishment and population persistence in suitable habitats (Cheplick 2023).

RA represents a key component of plant life history (Reekie 1999; Cheplick 2005, 2020) and information on how it changes with resources and accumulated biomass can provide insight into the adaptive significance of particular mating strategies. At the seed stage, under limited resources, allocation to CL reproduction is often favoured in perennial grasses (Campbell *et al.* 1983; Schoen 1984; Bell and Quinn 1987; Cheplick 2007). However, when conditions are favourable, allocation to CH can exceed that of CL (Cheplick and Clay 1989; Lerner *et al.* 2008). In addition, allocation to CH often exceeds that to CL when terminal panicle mass is included in the estimate of CH allocation (Fig. 5). Allocation of dry mass to CH seeds on a per-tiller basis in *D. compressa* sometimes equalled allocation to CL seeds in the edge habitat, but in the shady interior, allocation to CH seeds was lower than allocation to CL seeds in 3 of 5 years (Fig. 5). Note that, in both habitats, CL allocation was far less variable from year to year relative to CH allocation (Table 2), and over all years was not greatly reduced in the interior (reduction of allocation from edge to interior was 11.3 % for CL compared to 36.4 % for CH). This relative spatiotemporal stability of allocation to CL seeds underscores the importance of cleistogamy to reproductive fitness in this species.

### Allometry of chasmogamous and cleistogamous fecundity and allocation

Because much of the variation in seed production (i.e. fecundity) in herbaceous plants is often due to variation in the mass of individuals (Aarssen and Taylor 1992) or ramets (Shipley and Dion 1992), the present study aimed to determine how tiller mass affects the number of seeds made by chasmogamy and cleistogamy in *D. compressa*. Tiller mass was highly significant as a covariate in the analysis of fecundity (Table 1), but separate analyses by year revealed that it was mostly the number of axillary CL seeds that showed an increase with tiller mass (Fig. 2). When significant, tiller mass explained 35–55 % of the variation in CL fecundity, but in most years it did not significantly explain variation in CH fecundity. This contrasts with the few other studies that show an increase in chasmogamy with increasing plant size (Diaz and Macnair 1998) or tiller size (Cheplick 2007). However, in a study with the perennial herb *Oxalis montana* in which CH flowers are made early in the season (mid-June to July) while CL flowers are made later up to mid-September, the same pattern as found here for *D. compressa* was noted: CL fecundity was ‘dependent on plant size to a greater degree than CH flower production’ (Jasieniuk and Lechowicz 1987). The increased CL fecundity with tiller size in *D. compressa* is probably also due to the seasonal timing of axillary CL panicle maturation. The axillary CL panicles are found within the leaf sheaths of sequential phytomers along the flowering tiller, subsequent to terminal CH panicle maturation. The more phytomers a flowering tiller has, the greater number of axillary seeds is matured [see **Supporting Information—Table S4**]. For late-developing axillary panicles in the upper phytomers, the CL florets may still not be mature with seeds at the time of senescence in September. Although the flowering tiller with the terminal panicle is a determinate structure, the axillary panicle within the leaf sheath of a phytomer continues to grow until tiller senescence. Differential timing in the maturation of CH and CL seeds may also be a reason why the CL seed set was often lower than the CH seed set (Fig. 1A and B).

Although seed set and mass per CH or axillary CL seed were unaffected by tiller mass, RA based on the collective mass of seeds matured was negatively related to tiller mass. A negative relationship between RA and plant size is commonly reported in herbaceous perennials (Reekie 1998; Cheplick 2005), including 15 caespitose perennial grasses examined by Wilson and Thompson (1989). Positive *y*-intercepts of the allometric relationship of reproduction to size [see **Supporting Information—Table S3**] typically result in a decrease in RA with increasing size (Samson and Werk 1986; Reekie 1998). This may be due to the increasing supporting costs of accessory structures such as stems and leaves required by larger plants that act as allometric constraints on allocation (Reekie 1999). Because the number of phytomers is strongly correlated with tiller mass in *D. compressa*, the number and mass of axillary CL seeds produced along phytomer internodes are coupled to tiller mass. CH panicles are determinate and made at the terminal end of a flowering tiller early in the summer before vegetative mass has ceased to accumulate over the ensuing months; thus, larger tillers collected in September will be likely to show lower allocation to CH than smaller tillers.

### Role of the cleistogene

The cleistogene has long been recognized as a prominent feature of the reproductive system of *Danthonia* grasses (Chase 1918; Weatherwax 1928; Dobrenz and Beetle 1966; Campbell *et al.* 1983; Darbyshire 2003). It is a large caryopsis that forms from a single CL floret at the short, basal phytomer of a tiller, tightly enclosed by the leaf sheath. In *D. compressa*, the mass of the cleistogene was always far greater than the mass of axillary CL or CH seeds (Fig. 4). Although following disarticulation of a flowering tiller after it senesces in autumn, the axillary CL seeds at the higher phytomers could be scattered about and disperse to a limited extent (Dobrenz and Beetle, 1966), the basal cleistogene generally remains in place. Thus, the cleistogene could be important to the persistence of a population, especially under mammalian grazing pressure (Dyksterhuis 1945; Campbell *et al.* 1983), analogous to an axillary ‘bud bank’ in other perennial grasses that are not cleistogamous (Hendrickson and Briske 1997; Ott *et al.* 2019). The large mass of the cleistogene may also be important for seedling establishment and survival, as it is for the axillary CL seeds germinating under natural conditions (Cheplick 2023). Experiments have shown that, although axillary CL seeds show high germination outdoors in spring following cold, moist storage (Cheplick 2023), mature cleistogenes treated similarly do not germinate well under the same conditions (*unpublished results*). Thus, dormant cleistogenes in a *Danthonia* population might also function as a ground-level seed bank. Heavy CL seeds could be a significant component of fitness within established populations of cleistogamous species; their lack of dispersibility (philomatry) and genetic similarity to their maternal parents due to self-fertilization could have a role to play in adaptation to the maternal habitat in which they were produced (see Cheplick 2022).

It is intriguing that mass per CH or axillary CL seed was not significantly correlated with tiller mass while cleistogene mass was closely correlated with tiller mass in both habitats [see **Supporting Information—Fig. S3**]. This suggests that environmental conditions that facilitate plant growth to larger sizes, for example, adequate sunlight available along woodland edges, will result in heavier cleistogenes that again could improve population persistence in favourable habitats.

## Conclusions

Analysis of the patterns of CH and CL reproduction in the native perennial grass *D. compressa* showed spatial and temporal variation in the two reproductive modes over a 5-year period. Relative to CH reproduction, fecundity, seed mass and biomass allocation were less variable for CL reproduction from year to year. Axillary CL seeds and especially the large basal cleistogene were much heavier than CH seeds made on terminal panicles. Also, both axillary CL seed production and cleistogene mass increased with increasing tiller mass and matured later in the season than CH seeds. The undispersed CL seeds are likely to be important to seedling establishment within the maternal habitat and could function to maintain populations, especially along woodland edges where this species mostly occurs.

## Supporting Information

The following additional information is available in the online version of this article—

**Raw data.** All primary variables recorded for the five years of tiller collection in the two habitats is reported and the manner by which derived variables were calculated is indicated.

**Figure S1.** Dissected flowering tiller of *Danthonia compressa* separated into five phytomers from the lowermost (right) to the uppermost (left) bearing the terminal panicle. The cleistogene and axillary CL seeds of each phytomer are removed from the enclosing leaf sheath. Note that CH seeds have dispersed from the terminal panicle, leaving attached glumes. Tiller was collected from the interior habitat in September 2017.

**Figure S2.** Mean monthly maximum (A) and minimum (B) temperatures during the growing period from May to September for the 5 years of the study. Red symbols show the 20-year averages for comparison.

**Figure S3.** The mass of the cleistogene regressed onto tiller vegetative mass for flowering tillers from the edge (A) and interior habitats (B) over the 5 years of the study.

**Table S1.** Means ± SE across the 5 years of tiller collection for CH and axillary CL reproductive variables in the edge and interior habitats.

**Table S2. (**A) Monthly and long-term average precipitation (mm) in May through September for the five years of *Danthonia compressa* tiller collection (2017–21) and (B) deviation (mm) of annual monthly precipitation from the long-term average.

**Table S3.** Summary of significant standardized major axis regressions shown in Figure 3.

**Table S4.** Fecundity (mean ± SE) by CH and CL in relation to the number of phytomers in the flowering tillers of *Danthonia compressa* collected from the edge and interior habitats.

**Table S5.** ANCOVA of reproductive allocation using the collective mass of CH seeds plus panicle mass (arcsine, square-root transformed) as the measure of allocation to CH.

plad020_suppl_Supplementary_Tables

plad020_suppl_Supplementary_Figures

plad020_suppl_Supplementary_Data

## Data Availability

Data for all primary variables for the five years of tiller collection in the two habitats is in the supporting information raw data file.

## Notes

**Populations & Communities. Guest editor:** Jean Burns
