# Supplementary material for: Spatiotemporal variation of chasmogamy and cleistogamy in a native perennial grass: fecundity, reproductive allocation and allometry
Source: AoB Plants. 2023 Apr 29;15(3):plad020. doi: 10.1093/aobpla/plad020 (PMC10184453; doi:10.1093/aobpla/plad020)
Supplement: plad020_suppl_Supplementary_Tables [file plad020_suppl_supplementary_tables.pdf]

Table S1. Means ± SE across the five years of flowering tiller collection for CH and axillary CL variables in the edge and interior habitats.

Variable	Edge		Interior	
	CH	CL	CH	CL
Number of florets per tiller*	21.5 ± 0.7	18.2 ± 0.6	17.9 ± 0.7	18.1 ± 0.7
Seed set (%)	76.4 ± 5.6	74.0 ± 3.1	76.3 ± 6.2	65.0 ± 5.2
Fecundity (= seed number per tiller)*	16.6 ± 1.9	13.1 ± 1.0	13.2 ± 2.3	12.2 ± 1.4
Mass (mg) per seed	0.70 ± 0.03	1.16 ± 0.02	0.63 ± 0.02	1.05 ± 0.05
Mass (mg) per cleistogene*	–	2.53 ± 0.16	–	2.07 ± 0.19
Allocation to seeds (%)	9.4 ± 0.8	10.6 ± 0.4	8.2 ± 0.6	11.1 ± 0.5
Allocation to CH seeds *plus* terminal panicles(%)	15.3 ± 1.0	–	14.3 ± 0.8	–

*Least-squares means reported for these variables in which the effect of vegetative tiller mass as a covariate was significant in an ANCOVA.

Table S2. (a) Monthly and long-term average precipitation (mm) in May through September for the five years of *Danthonia compressa* tiller collection (2017-2021) and (b) deviation (mm) of annual monthly precipitation from the long-term average.

Year	May	Jun	Jul	Aug	Sep
(a) Precipitation (mm)					
1	137.9	154.7	129.8	65.3	49.0
2	66.8	80.0	120.4	152.6	216.9
3	126.5	143.3	74.2	115.6	40.4
4	77.2	82.5	98.8	219.5	61.5
5	105.2	48.8	282.9	96.8	103.4
Average	91.7	109.5	107.9	90.7	87.1
(b) Deviation from long-term average (mm)					
1	+46.2	+45.2	+21.9	-25.4	-38.1
2	-24.9	-29.5	+12.5	+61.9	+129.8
3	+34.8	+33.8	-33.7	+24.9	-46.7
4	-14.5	-27.0	-9.1	+128.8	-25.6
5	+13.5	-60.7	+175.0	+6.1	+16.3

Chasmogamous seeds are mature by mid-July, while basal and axillary cleistogamous seeds are mature by early September.

Table S3. Summary of significant standardized major axis regressions shown in Figure 2.

Year	Seed type	F	r^2	$\alpha_{RMA} \pm SE$	$\log\beta_{RMA} \pm SE$	Figure
1	CL	16.6***	0.37	1.11 ± 0.17	0.98 ± 0.35	2a
3	CL	14.3***	0.35	0.88 ± 0.14	1.08 ± 0.28	2e
	CH	19.4***	0.41	1.06 ± 0.15	1.14 ± 0.32	2f
5	CL	34.5***	0.55	0.71 ± 0.09	1.26 ± 0.19	2i

α_{RMA} and β_{RMA} are the slope and *y*-intercept, respectively.

***$P < 0.001$.

Table S4. Fecundity (mean ± SE) by CH and CL in relation to the number of phytomers in the flowering tillers of *Danthonia compressa* collected from the edge and interior habitats.

	Edge			Interior		
Number of phytomers	Number of tillers	CH	CL	Number of tillers	CH	CL
4	6	21.7 ± 4.3	13.1 ± 2.1	7	6.9 ± 1.4	10.3 ± 1.4
5	37	16.4 ± 1.2	12.9 ± 0.9	40	11.6 ± 1.0	9.4 ± 0.5
6	24	17.1 ± 1.4	16.3 ± 1.2	26	15.0 ± 1.6	12.8 ± 1.1
7	8	17.9 ± 3.5	17.4 ± 2.1	2	24.5	22.5
F_{PHY}		0.12	5.21*		14.78***	6.08**

"Number of tillers" is the number of flowering tillers collected with the number of phytomers indicated over five successive years (2017-2021). F_{PHY} is from a regression of fecundity onto the number of phytomers for each seed type in each habitat.

*$P < 0.05$; **$P < 0.01$, ***$P < 0.001$.

Table S5. ANOVA of reproductive allocation using the collective mass of CH seeds plus panicle mass (arcsine, square-root transformed) as the measure of allocation to CH in flowering tillers of *Danthonia compressa*.

Source of variation	d.f.	M.S.	*F*
Year	4	0.0432	7.71***
Habitat	1	0.0003	0.06
Type	1	0.2060	36.82***
Year × habitat	4	0.0249	4.46**
Year × type	4	0.0851	15.21***
Habitat × type	1	0.0065	1.16
Year × habitat × type	4	0.0115	2.06
Error	279	0.0056	

Flowering tillers were from woodland edge or interior habitats in five successive years (2017-2021). "Type" refers to allocation to CH seeds plus panicle mass vs. allocation to CL seeds.

*$P < 0.05$; **$P < 0.01$, ***$P < 0.001$.
