# Supplementary material for: Spatiotemporal variation of chasmogamy and cleistogamy in a native perennial grass: fecundity, reproductive allocation and allometry
Source: AoB Plants. 2023 Apr 29;15(3):plad020. doi: 10.1093/aobpla/plad020 (PMC10184453; doi:10.1093/aobpla/plad020)
Supplement: plad020_suppl_Supplementary_Figures [file plad020_suppl_supplementary_figures.pdf]

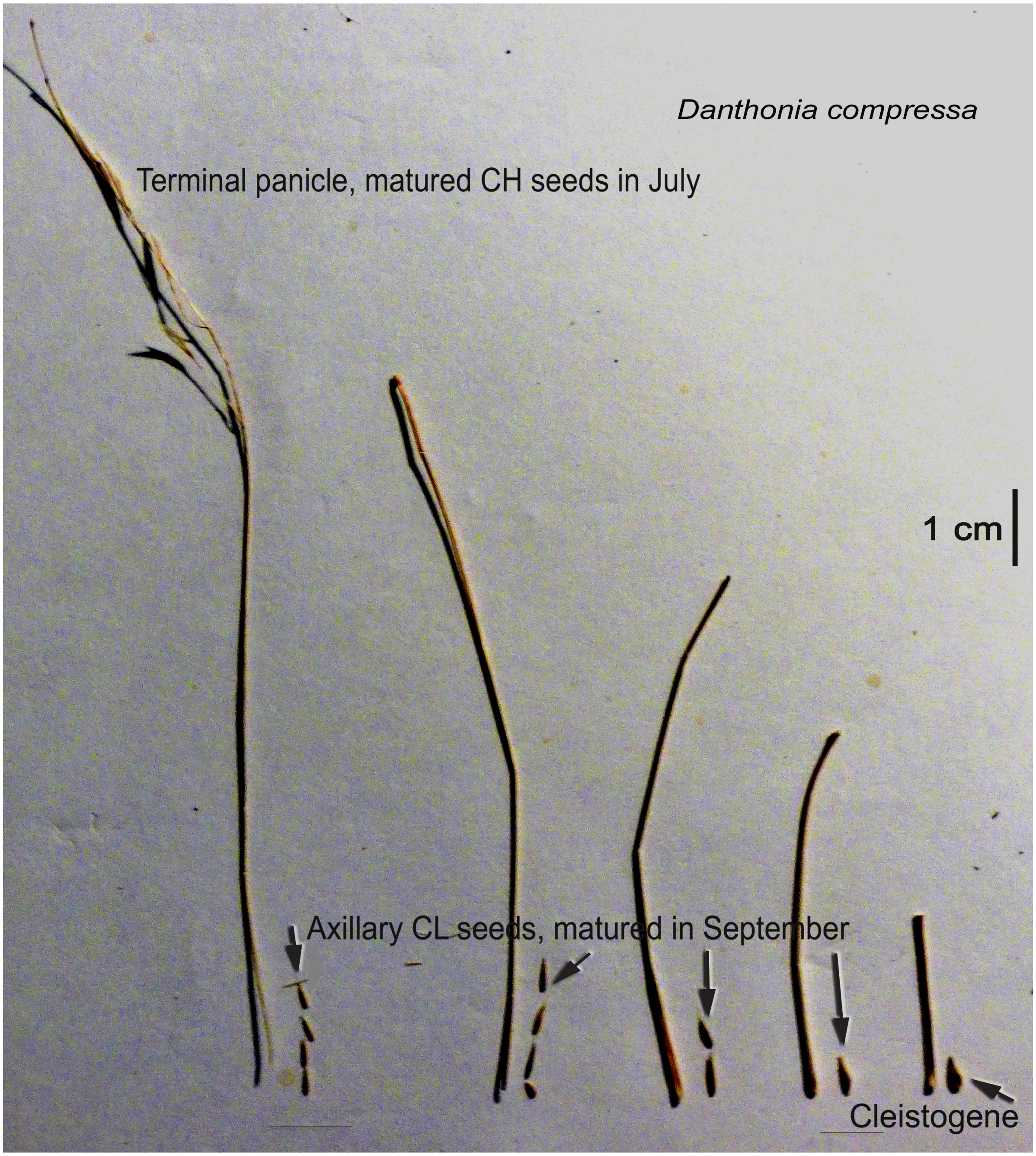

Figure S1. Dissected flowering tiller of *Danthonia compressa* separated into five phytomers from the lowermost (right) to the uppermost (left) bearing the terminal panicle. The cleistogene and axillary CL seeds of each phytomer are removed from the enclosing leaf sheath. Note that CH seeds have dispersed from the terminal panicle, leaving attached glumes. Tiller was collected from the interior habitat in September, 2017.

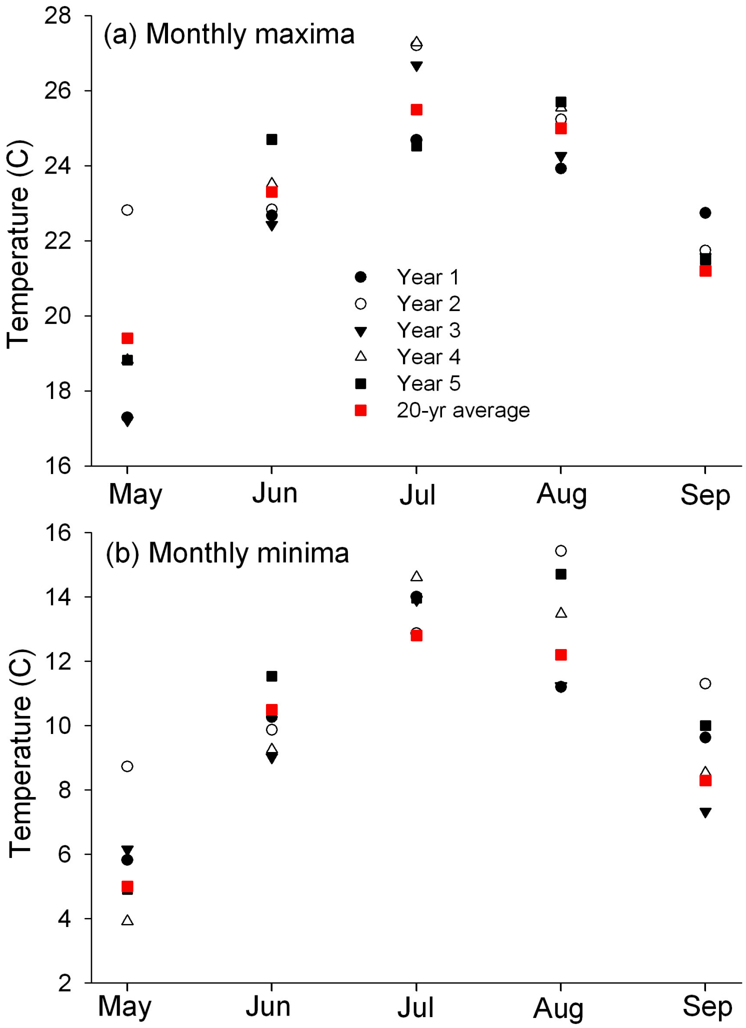

Figure S2. Mean monthly maximum (a) and minimum (b) temperatures during the growing period from May to September for the 5 yrs of the study. Red symbols show the 20-yr averages for comparison.

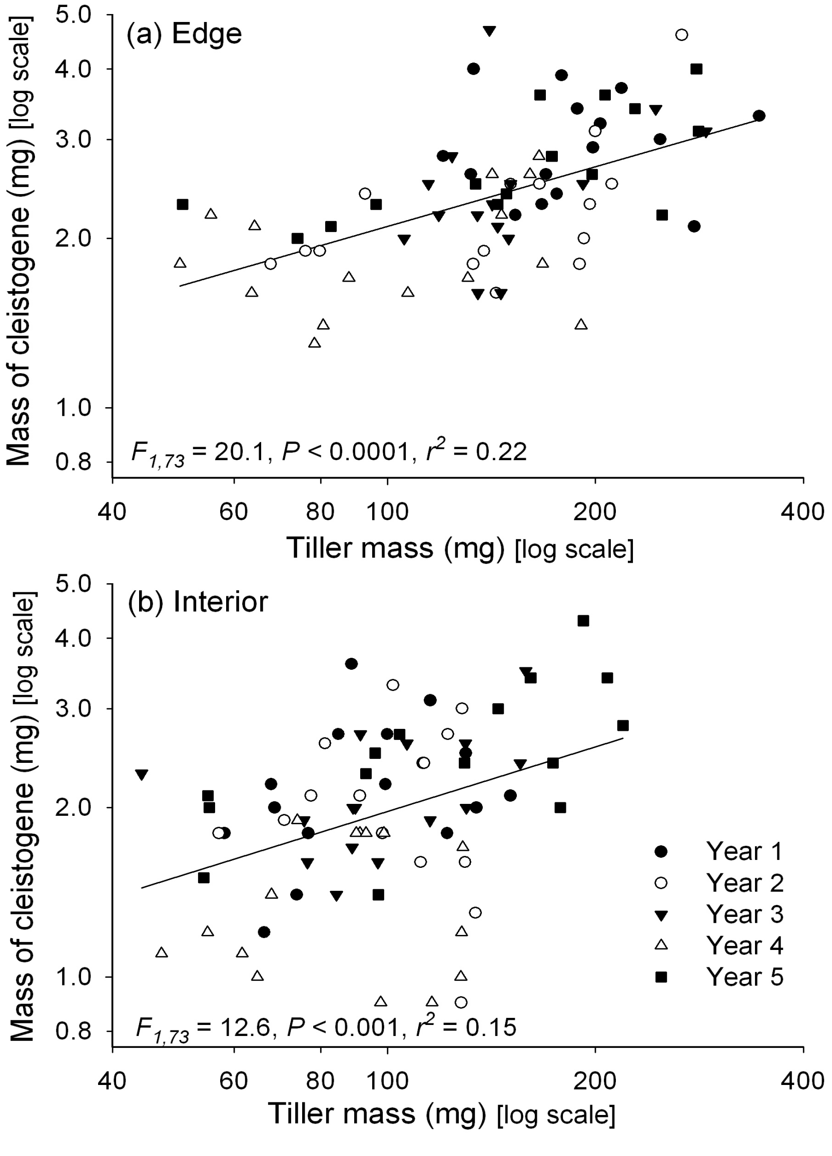

Figure S3. The mass of the cleistogene regressed onto tiller vegetative mass for flowering tillers from the edge (a) and interior habitats (b) over the 5 yrs of the study.
