# Supplementary material for: Spatiotemporal variation of chasmogamy and cleistogamy in a native perennial grass: fecundity, reproductive allocation and allometry
Source: AoB Plants. 2023 Apr 29;15(3):plad020. doi: 10.1093/aobpla/plad020 (PMC10184453; doi:10.1093/aobpla/plad020)
Supplement: plad020_suppl_Supplementary_Data [file plad020_suppl_supplementary_data.pdf]

These are the raw data for the primary variables in “Spatiotemporal variation of chasmogamy and cleistogamy in a native perennial grass: fecundity, reproductive allocation and allometry” by G.P. Cheplick [Professor Emeritus, Biology Program, Plant Science Subprogram, City University of New York, New York, NY 10016]

Fifteen tillers/habitat/year were sampled from individual *Danthonia compressa* in each of five successive years from a sunny edge [EDGE] & an adjacent woodland interior [INT] habitat [HAB]. Coding is for Version 9.4, SAS University Edition.

<u>Definition of primary variables</u>: Note—all are values per each collected flowering tiller. A flowering tiller has a single terminal panicle with CH spikelets, axillary CL panicles in the leaf sheaths along each phytomer, plus a single basal cleistogene in the lowermost phytomer.

PHYTOS = number of phytomers
TDM = tiller vegetative dry mass (mg) (*i.e.*, the mass of culm plus leaves)
NOCL = number of axillary CL seeds
CLEMPTY = number of empty axillary CL florets
AXCLMA = collective dry mass (mg) of axillary CL seeds
BACL = dry mass (mg) of the single basal cleistogene
NOCHSP = number of CH spikelets per terminal panicle
NOCH = number of CH seeds per terminal panicle
CHEMPTY = number of empty CH florets per terminal panicle
CHMA = collective dry mass (mg) of CH seeds
APM = dry mass (mg) of the aerial (terminal) panicle without the attached CH spikelets & seeds

<u>Definition of derived variables:</u>

ONEAXCL = mass per axillary CL seed
ONECH = mass per CH seed
TCLMA = total mass of axillary CL seeds plus basal cleistogene
SETAXCL = proportion of seed set in axillary CL florets
SETCH = proportion of seed set in CH florets
PROCL = proportion of total fecundity due to CL reproduction
ALLOCL = reproductive allocation to CL seeds (including the cleistogene)
ALLOCH1 = reproductive allocation to CH seeds
ALLOCH2 = reproductive allocation to CH seeds plus the terminal panicle

```
DATA DANTH;

INPUT YEAR HAB$ PHYTOS TDM NOCL CLEMPTY AXCLMA BACL NOCHSP NOCH
CHEMPTY CHMA APM;

ONEAXCL=AXCLMA/NOCL; ONECH=CHMA/NOCH; TCLMA=AXCLMA+BACL;

SETAXCL=NOCL/(NOCL+CLEMPTY); SETCH=NOCH/(NOCH+CHEMPTY);
PROCL=NOCL/(NOCL+NOCH);

ALLOCL=(AXCLMA+BACL)/TDM; ALLOCH1=CHMA/TDM;
ALLOCH2=(CHMA+APM)/TDM;

DATALINES;
1 EDGE 4 198.35 18.0 4.0 18.45 2.9 8 25 6 22.5 10.9
1 EDGE 4 120.45 12.5 4.5 12.20 2.8 9 35 10 33.2 13.2
```

1 EDGE 4 169.55 21.0 7.0 17.10 2.6 10 28 9 27.0 15.6
1 EDGE 5 345.05 17.0 9.5 21.40 3.3 7 16 8 12.5 8.5
1 EDGE 5 203.35 16.5 6.5 15.90 3.2 8 13 6 14.6 10.9
1 EDGE 5 248.10 19.5 8.5 20.90 3.0 9 18 9 12.4 13.2
1 EDGE 5 188.20 17.5 8.0 15.45 3.4 10 19 15 16.7 15.6
1 EDGE 5 175.85 13.0 10.0 12.85 2.4 8 23 13 15.8 10.9
1 EDGE 5 167.25 11.0 8.5 9.35 2.3 8 19 13 15.9 10.9
1 EDGE 5 218.10 15.0 3.0 15.45 3.7 7 5 10 3.7 8.5
1 EDGE 6 278.10 15.0 4.0 12.80 2.1 9 20 9 11.8 13.2
1 EDGE 5 178.75 15.0 2.0 16.55 3.9 7 16 10 11.1 8.5
1 EDGE 5 153.15 9.5 7.0 8.75 2.2 7 8 9 5.8 8.5
1 EDGE 5 133.25 13.5 3.0 12.40 4.0 6 4 8 2.9 6.2
1 EDGE 5 132.00 12.0 4.5 9.75 2.6 7 11 9 7.3 8.5
1 INT 5 150.75 8.0 6.5 8.95 2.1 7 6 14 4.8 8.4
1 INT 5 68.65 7.0 7.0 6.10 2.0 5 4 10 2.5 4.7
1 INT 5 73.90 8.0 6.0 5.30 1.4 7 15 6 13.8 8.4
1 INT 5 84.90 9.0 7.0 5.50 2.7 5 9 5 5.0 4.7
1 INT 5 129.85 8.5 4.5 8.55 2.5 7 20 4 15.4 8.4
1 INT 6 122.00 9.0 3.0 9.80 1.8 9 31 7 24.2 12.1
1 INT 5 67.85 8.5 5.5 6.30 2.2 6 13 6 11.9 6.5
1 INT 5 99.95 8.0 7.5 6.75 2.7 6 4 7 2.5 6.5
1 INT 5 66.35 13.0 7.0 10.35 1.2 8 15 7 11.1 10.2
1 INT 5 115.40 10.0 6.5 9.70 3.1 6 10 5 6.0 6.5
1 INT 5 58.10 6.0 7.5 6.35 1.8 7 12 8 9.1 8.4
1 INT 5 134.50 11.0 6.0 7.90 2.0 4 4 10 2.5 2.8
1 INT 5 99.30 2.0 12.0 1.50 2.2 6 9 7 4.7 6.5
1 INT 6 88.70 3.0 15.0 3.00 3.6 7 14 6 7.6 8.4
1 INT 6 76.80 11.0 6.0 6.90 1.8 6 10 6 6.0 6.5

2 EDGE 6 192.5 9 4 9.8 2.0 6 11 7 8.8 6.2
2 EDGE 6 199.8 23 7 20.8 3.1 7 26 9 18.3 8.5
2 EDGE 5 67.8 9 6 6.5 1.8 6 21 3 15.2 6.2
2 EDGE 5 133.1 13 10 8.7 1.8 10 19 5 15.8 15.6
2 EDGE 5 211.2 19 6 17.2 2.5 7 6 3 4.3 8.5
2 EDGE 5 143.7 6 2 3.8 1.6 8 32 2 28.6 10.9
2 EDGE 6 150.8 6 0 7.9 3.6 7 17 5 13.0 8.5
2 EDGE 7 266.6 22 4 24.2 4.6 8 36 2 29.2 10.9
2 EDGE 6 137.9 15 2 14.5 1.9 9 24 4 15.9 13.2
2 EDGE 6 196.4 11 1 14.2 2.3 8 25 2 17.4 10.9
2 EDGE 7 189.7 11 2 11.5 1.8 8 22 1 14.3 10.9
2 EDGE 6 166.0 15 2 14.8 2.5 6 12 2 7.4 6.2
2 EDGE 5 92.8 14 3 11.3 2.4 7 29 0 16.9 8.5
2 EDGE 5 79.8 14 0 13.8 1.9 7 12 5 8.7 8.5
2 EDGE 5 76.1 13 3 9.0 1.9 7 15 1 10.0 8.5
2 INT 5 111.7 8 6 6.8 1.6 5 17 0 13.2 4.7
2 INT 5 91.2 7 3 4.8 2.1 6 17 1 12.6 6.5

```
2 INT 5 112.5 10 5 8.1 2.4 7 19 2 11.9 8.4
2 INT 6 127.8 12 4 6.3 0.9 6 14 2 11.7 6.5
2 INT 5 134.1 9 5 7.3 1.3 6 14 2 9.3 6.5
2 INT 6 113.0 13 6 11.0 2.4 8 19 2 14.0 10.2
2 INT 5 57.0 10 2 7.9 1.8 6 12 0 7.2 6.5
2 INT 6 98.3 11 4 10.1 1.8 7 13 3 8.7 8.4
2 INT 5 77.5 9 3 7.1 2.1 6 24 5 17.5 6.5
2 INT 6 81.2 10 4 9.2 2.6 6 10 4 6.5 6.5
2 INT 5 129.5 9 3 9.5 1.6 8 12 2 7.9 10.2
2 INT 6 101.9 14 4 11.7 3.3 5 13 1 7.6 4.7
2 INT 5 128.3 10 2 7.9 3.0 9 19 1 11.9 12.1
2 INT 6 122.3 13 5 13.5 2.7 6 14 5 8.0 6.5
2 INT 6 70.9 5 2 3.8 1.9 5 8 0 4.2 4.7

3 EDGE 5 244.3 20 4 20.9 3.4 6 10 1 7.1 11.0
3 EDGE 5 114.6 14 7 11.9 2.5 6 9.5 2 7.3 6.2
3 EDGE 6 288.8 27 7 30.9 3.1 6 11 1 7.1 8.5
3 EDGE 5 118.6 19 13 16.2 2.2 7 9 1 7.2 6.8
3 EDGE 5 105.7 21 1 14.3 2.0 6 8 4 4.7 6.4
3 EDGE 5 149.7 13 11 11.6 2.0 8 11 0 10.8 8.1
3 EDGE 6 135.0 21 0 16.6 2.2 8 19 1 12.2 5.7
3 EDGE 5 135.2 . . 1.5 1.6 8 11 6 3.7 8.8
3 EDGE 7 141.8 25 0 21.3 2.3 7 13 0 10.6 5.0
3 EDGE 6 140.4 14 1 13.6 4.7 7 9 0 7.2 4.6
3 EDGE 6 145.9 12 0 9.5 1.6 6 12 1 8.0 3.6
3 EDGE 6 144.3 11 2 9.8 2.1 7 8 0 6.5 3.5
3 EDGE 5 123.9 12 2 12.6 2.8 8 10 1 7.6 3.8
3 EDGE 6 191.8 20 7 19.1 2.5 7 9.5 1 7.0 4.7
3 EDGE 6 150.8 18 6 14.8 2.5 6 7 0 5.6 4.5
3 INT 6 129.6 17 3 13.7 2.6 6 6 1 3.6 3.1
3 INT 4 89.9 11 4 8.6 2.0 5 5 1 3.5 3.0
3 INT 6 155.6 11 8 9.5 2.4 7 7 2 3.7 4.8
3 INT 4 88.9 14 1 10.5 1.7 7 6 1 3.5 5.1
3 INT 6 130.0 17 9 9.3 2.0 7 7 1 3.9 3.8
3 INT 5 44.1 11 2 8.0 2.3 3 4 0 2.2 0.9
3 INT 6 115.3 15 1 10.9 1.9 5 7 0 4.6 2.3
3 INT 5 91.3 13 5 9.9 2.7 6 7 1 3.3 2.5
3 INT 5 75.7 12 5 8.4 1.9 5 6 2 3.8 1.6
3 INT 6 158.5 10 4 7.2 3.5 7 8 0 5.0 2.8
3 INT 5 96.8 10 2 9.7 1.6 5 6 1 3.6 1.6
3 INT 6 76.6 8 3 5.7 1.6 5 4 1 2.0 2.7
3 INT 4 89.2 9 7 7.1 2.0 6 3 1 2.2 3.2
3 INT 5 106.7 13 8 9.2 2.6 7 5 1 2.7 3.4
3 INT 5 84.4 8 4 6.8 1.4 5 8 2 4.8 2.5
```

4 EDGE 5 146.2 10 15 11.0 2.2 8 10 12 4.6 11.8
4 EDGE 5 80.7 5 8 4.6 1.4 8 19 7 10.8 8.9
4 EDGE 5 107.0 10 11 6.3 1.6 6.5 22 2 20.9 7.5
4 EDGE 5 165.6 16 4 16.8 2.8 8 20 9 14.9 9.2
4 EDGE 5 87.9 16 5 9.4 1.7 5 27 7 18.7 5.3
4 EDGE 5 167.6 5 12 4.6 1.8 10 15 2 9.8 10.4
4 EDGE 7 141.7 9 3 11.5 2.6 5 13 8 6.6 6.5
4 EDGE 5 64.2 2 1 0.8 4.1 4 19 1 11.9 4.3
4 EDGE 6 130.7 10 2 10.9 1.7 7 29 1 23.3 8.4
4 EDGE 7 160.7 13 5 9.7 2.6 6 18 6 13.3 5.3
4 EDGE 5 55.5 4 11 3.8 2.2 6 26 3 19.2 5.3
4 EDGE 5 63.6 7 5 5.7 1.6 6 24 4 12.7 4.9
4 EDGE 6 190.6 15 2 14.2 1.4 8 24 5 16.0 13.1
4 EDGE 5 78.4 10 5 8.0 1.3 6 18 4 12.0 4.8
4 EDGE 4 50.1 9 1 7.9 1.8 6 18 2 11.6 9.8
4 INT 5 116.0 13 7 10.8 0.9 7.8 6 8 3.0 9.9
4 INT 5 97.7 5 15 4.1 0.9 6 8 11 3.5 6.6
4 INT 5 128.5 8 8 8.1 1.7 8.7 11 5 6.2 11.5
4 INT 5 91.2 11 12 8.5 1.8 6 11 1 5.7 4.2
4 INT 6 127.7 3 14 4.1 1.0 8.6 8 6 4.2 11.4
4 INT 5 67.9 6 10 4.4 1.4 6 6 2 3.9 4.4
4 INT 4 54.9 6 2 4.2 1.2 3 8 5 4.9 1.8
4 INT 5 90.1 4 3 3.3 1.8 6 6 3 3.3 6.6
4 INT 5 61.6 8 7 5.0 1.1 4 12 6 6.3 2.7
4 INT 6 64.8 15 13 10.8 1.0 5 17 16 11.2 4.2
4 INT 5 93.1 11 8 9.0 1.8 6 20 3 12.3 5.7
4 INT 5 98.8 7 5 6.0 1.8 8 14 9 10.4 11.3
4 INT 6 128.0 7 10 7.2 1.2 8.6 16 6 9.2 11.4
4 INT 4 47.1 5 2 3.7 1.1 2 4 2 2.4 1.0
4 INT 5 74.1 9 7 6.5 1.9 4 7 5 4.0 4.9

5 EDGE 6 148.7 14 10 13.9 2.4 7 24 2 16.6 6.8
5 EDGE 6 96.3 10 5 7.3 2.3 6 17 2 9.9 5.7
5 EDGE 7 134.1 15 5 13.4 2.5 6 3 17 1.0 8.6
5 EDGE 6 166.2 18 14 15.5 3.6 7 11 9 6.2 6.1
5 EDGE 4 82.8 10 4 8.6 2.1 7 20 3 14.7 8.6
5 EDGE 5 74.1 9 2 7.5 2.0 7 25 5 15.1 5.6
5 EDGE 5 197.8 25 3 21.2 2.6 8 28 6 14.1 7.9
5 EDGE 6 172.9 18 4 15.9 2.8 7 16 12 8.0 6.8
5 EDGE 6 206.3 18 7 18.8 3.6 8 20 8 7.5 7.6
5 EDGE 6 228.2 17 2 17.9 3.4 8 25 2 16.2 5.2
5 EDGE 7 144.3 22 6 19.4 2.3 7 25 2 14.5 7.0
5 EDGE 6 282.0 23 7 23.9 3.1 9 7 20 3.9 12.8
5 EDGE 4 50.5 8 2 7.6 2.3 5 4 12 4.0 3.6
5 EDGE 6 249.8 32 2 23.5 2.2 8 26 7 16.6 8.1
5 EDGE 7 280.0 22 5 24.9 4.0 11 13 20 7.1 10.6

```
5 INT 7 173.5 21 6 20.3 2.4 8 32 5 19.7 9.0
5 INT 5 104.1 14 5 12.1 2.7 6 16 4 8.5 4.7
5 INT 6 97.0 15 5 11.5 1.4 6 18 1 11.4 5.3
5 INT 6 96.0 16 4 9.9 2.5 7 14 10 9.0 5.8
5 INT 6 192.0 18 7 16.9 4.3 9 24 9 16.4 7.9
5 INT 6 161.1 30 9 26.0 3.4 6 28 3 19.4 6.5
5 INT 6 219.1 23 4 20.1 2.8 7 28 0 19.3 6.0
5 INT 7 177.9 24 8 18.3 2.0 9 17 2 13.3 7.7
5 INT 5 54.2 8 6 5.9 1.5 6 11 0 7.3 3.7
5 INT 5 55.0 16 7 11.6 2.1 4 10 2 4.8 3.0
5 INT 4 55.2 14 8 9.7 2.0 4 14 0 8.3 3.9
5 INT 5 129.3 17 4 15.9 2.4 8 34 2 24.2 7.1
5 INT 4 93.1 13 4 11.9 2.3 7 8 14 3.8 9.0
5 INT 6 208.0 12 9 12.3 3.4 9 34 2 23.0 8.8
5 INT 6 144.5 16 2 13.0 3.0 8 18 9 10.8 10.2
;
```
